# Spatial distribution and radiological hazards assessment of naturally occurring radionuclide materials in soil from quarry sites in Ogun State, Nigeria

**DOI:** 10.1007/s10661-025-13988-6

**Published:** 2025-04-22

**Authors:** David O. Jegede, T. Adeniyi Afolabi, Foluso O. Agunbiade, T. Adeleke Afolabi, Olusegun O. Ogundiran, Muideen R. Gbadamosi, Samuel O. Sojinu, Oluseyi Z. Ojekunle, Pakorn Varanusupakul

**Affiliations:** 1 Department of Basic Sciences (Chemistry Unit), Babcock University, Ilishan-Remo, Nigeria; 2 Division of Environmental Health Sciences, College of Public Health, The Ohio State University, Columbus, OH 43210 USA; 3 Department of Chemistry, Federal University of Agriculture, Abeokuta, Nigeria; 4 Department of Chemistry, University of Lagos, Akoka, Nigeria; 5 Department of Laboratory Services, Nigerian Institute of Science Laboratory Technology, Ibadan, Nigeria; 6 Department of Chemistry, Sikiru Adetona College of Education, Omu-Ijebu, Ogun, Nigeria; 7 School of Geography Earth and Environmental Science, University of Birmingham, Birmingham, UK; 8 Department of Environmental Management and Toxicology, Federal University of Agriculture, Abeokuta, Nigeria; 9 Department of Chemistry, Chulalongkorn University, Bangkok, Thailand

**Keywords:** Radiological risk, Quarry, Ogun State, Radioactive, Gamma-ray spectrometry

## Abstract

Workers and dwellers around quarrying sites are exposed to naturally occurring radioactive materials (NORMs) during various activities done on the rock and earth crust. This study investigated the spatial distribution and radiological health effects of quarrying activities in ten quarry sites in three districts (Odeda, Ajebo, and Ijebu Ode) around Ogun State, Nigeria. The NORMs (^40^K, ^238^U, ^232^Th) were assessed using a gamma spectrometer with a NaI(Tl) detector. The radiological hazards of NORMs were assessed and statistically analyzed. The activity concentration of NORMs (Bq/kg) ranged from ^40^K (76.8 ± 44.8–2647.9 ± 179.4), ^238^U (3.2 ± 1.8–55.4 ± 24.9), and ^232^Th (5.2 ± 3.9–244.4 ± 89.8) revealing 70% of all samples above the world average limit 420(^40^K), 33(^238^U), and 45 (^232^Th). The activity concentration of NORMs in all the sites followed in the order ^238^U < ^232^Th < ^40^K. The radiological and health parameter ranges for the adsorbed dose rate (D_R_) 3.0–339.92 (nGy/h), radium equivalent (Ra_eq_) 5.88–739.4 (Bq/kg), annual effective dose equivalent outdoor (AEDEout) 3.72–417.16(µSvy^−1^), excess lifetime cancer risk (ELCR × 10^−3^) 0.01–1.46, and exposure rate (ER) 13.10–1531.47(µRh^−1^). The radiological hazard parameters are 2–3 times higher than their world averages in most of the samples thus discouraging the usage of the soil for building and ecological activities. This study showed that radionuclides are priority pollutants with high impact and with high exposure risk tendencies in all the quarry sites investigated and therefore unsuitable for ecological and building activities.

## Introduction

Soils and rocks serve as the primary sources of terrestrial radiation, as volcanic formations and rocks rich in phosphate, granite, and salt naturally contain radionuclides such as ^226^Ra, ^238^U, ^40^K, and ^232^Th (Abbasi, 2022: Regassa et al., 2023; Maglas et al., 2025). These radionuclides considerably contribute to the natural radiation that is found outdoor on land (Abbasi et al., 2023; Isinkaye et al., 2023; Khalaf et al., 2025). Approximately 96% of the total radiation exposure in humans originates from natural sources, while only 4% comes from artificial sources (Tawfic et al., 2021). Gamma emitting radiations such as ^238^U, ^40^K, and ^232^Th contribute more to the external exposure while ^226^Ra is responsible for the 98% of ^238^U decay subseries and it is occasionally regarded as of ^238^U (Regassa et al., 2023). The uranium- 238 series, which starts with ^238^U and decays through various intermediate isotopes like radium- 226 and polonium- 210, eventually reaching stable lead- 206 (^206^Pb), plays a crucial role in the radiological assessment. Similarly, the thorium- 232 series, beginning with ^232^Th, progresses through radium- 228, actinium- 228, and thoron (radon- 220) before ending with stable lead- 208 (^208^Pb) (Liu et al., 2021; Zhang et al., 2022). Rock quarrying and the crushing of stone is a global phenomenon which has been one of the major environmental concerns everywhere around the world, including the developed countries because quarries contribute to soil contamination, air pollution, and negative impacts on natural resources and they are accountable for the significant amounts of dust and other pollutants released into the atmosphere, which have a detrimental effect on the air, soil, and other ecosystems (Ambastha & Haritash, 2022). Quarrying operations have resulted in considerable and irreversible changes to ecosystems and biological connections (Waweru et al., 2018). Quarrying activities in Ogun State play a significant role in the economic development of the state. They provide essential construction materials, generate government revenue through taxes and royalties, and create employment opportunities, particularly for the rural population (Jegede et al., 2025). In the process of quarry activities and mining of rocks, overburden is removed, stones are drilled and cut, and occasionally rocks are blasted and crushed (Ofomola et al., 2023; Waweru et al., 2018). The ecology and the socioeconomic well-being of the locals living in the vicinity of quarried regions are impacted by the operation of the quarry and the landscape scars left by quarry activities. (Misthos et al., 2018). Since the beginning of time, the primordial radioactive ^40^K (potassium), ^232^Th (thorium), and ^238^U (uranium) have existed on Earth. These series are significant for tracking radiation exposure in humans (Gbadamosi et al., 2018; UNSCEAR, 2000). According to Ibikunle et al. (2019) and Duong et al. (2021), plants have the ability to absorb radioactive from soil and transmit them to humans through edible plants, making it easy for them to accumulate in the food chain. Natural radioactive elements possess the capacity to build up in bodily organs, endangering the digestive and endocrine systems. According to Biira et al. (2021) and Kefalati et al. (2021), there is evidence of uranium build-up in the kidneys, lungs, and thyroid gland, as well as potassium build-up in the human muscles. Naturally occurring radioactive material (NORMs) decay can often result in the emission of beta, alpha, and gamma radiation into the body’s organs. The emitted radiation particles have the capacity to injure these essential organs, making them cancerous (Biira et al., 2021; Saenboonruang et al., 2018). As such, a lot of focus has been on evaluating the radiation risks and measuring the activity concentration of naturally occurring radioactive elements in quarry products such as soil and rock (Duong et al., 2021; Nduka et al., 2022). Radionuclides in soil become a radiological health risk when their activity concentrations are above a certain permitted threshold. Therefore, it has been established that exposure to them raises the chance of leukemia in addition to several other cancers, such as the kidney, prostate, and melanoma (Bangotra et al., 2018). Instability in the atoms of primordial radionuclides in the environment causes radioactivity, which is pervasive in the environment and harmful to human health (Arıman & Gümüş, 2018). There has been a paucity of research on NORMS in regions with elevated natural background radiation and areas with commercial interests, such as the maritime environment, soil (Ahmad et al., 2015; Gbadamosi et al., 2018), and mining (Khattab et al., 2024; Onjefu et al., 2025). ^238^U, ^232^Th, and ^40^K, have average activity concentrations of 11.5 ± 1.0–166 ± 40 Bq/kg, 15.6 ± 1.8–31.4 ± 2.3 Bq/kg, and 20.4 ± 1.3–366 ± 30 Bq/kg, respectively, according to research by Gbadamosi et al. (2017) done on sediments of waste dumpsites in Nigeria. It was discovered that the radiological parameters in two of the sampling locations were greater than the average value (Bq/kg) worldwide of 420(^40^K), 33(^238^U), and 45 (^232^Th). Unfortunately, the carcinogenic and radioactive dangers linked to exposure to NORMs in quarry soils in Ogun State are generally not understood yet. Thus, this study aims to assess the radiological risks related to radioactive materials detected in soil around quarry sites in Ogun State.

## Materials and methods

### Study area

**Fig. 1:**
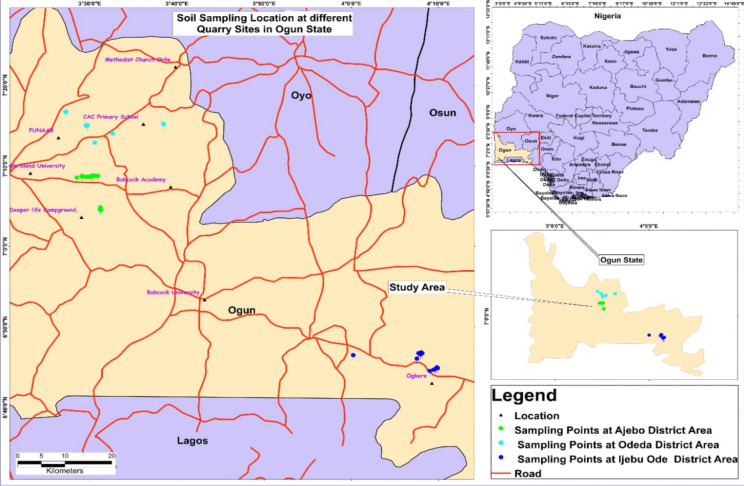
Map showing the sampling points in the quarry sites in all district areas in Ogun State, Nigeria

The study was conducted in Ogun State, which has a population of 3,751,140, a land mass area of 16,980.55 km^2^, a density of 220/km^2^, and coordinates of 3.4737° E and 6.9980° N (Olukanni et al., 2020). A total of ten (10) selected quarry sites approximately 1.5 to 7 km apart from each other were selected for the study (Fig. 1). The quarry site names (code) are Skelly (SKY), S and D (S&D), Alabata (ALA), Shogor (SHG), (Odeda Local Government Area), Ratcon (RAT), Safe (SAF) (Ijebu Ode Local Government Area), Abl (ABL), Labstar (LAB), 24Hrs (24H), and Hoy (HOY) (Obafemi Owode Local Government Area).

### Sampling and sample preparation

A total of two hundred and fifty (250) soil samples of 1 kg each were collected at ten (10) quarry sites in Ogun State. Using a soil auger, the soils were sampled at a depth of 0–15 cm and transferred in a polyethylene bags along with one control sample each (site with no anthropogenic input from the quarry sites). To compare the concentrations of pollutants in the quarry site(s) and the dwelling area, the control representing each area was brought together. After being transported, the samples were prepared at the laboratory. The twenty-five (25) soil samples from each quarry site were thoroughly mixed for uniformity. Using the coning and quartering method, the bulk sample was repeatedly divided and reduced while maintaining representativeness. The final homogenized sample was then split into five equal portions, yielding five representative samples per site, and were properly labeled for identification. After being sun-dried and ground into a powder using mortar and pestle, the representative samples were sieved through a 0.2-mm mesh screen to get rid of lumps, pebbles, and organic materials. After that, the samples were kept in the laboratory for further analysis. Throughout the sample procedure, personal protective equipment was worn to ensure appropriate protection. To avoid identification errors, codes were applied to the soil samples that were gathered from each site. Coordinates were taken for each of the sampling points (quarry sites) including control sites using a global positioning system (GPS).

### Naturally occurring radionuclide analysis

#### Radioactivity measurement and counting

Using a gamma ray spectrometer (GS- 2000-Pro) with a detector NaI (Tl) 3 × 3-inch, Princeton Gamma Tech (USA), on the soil samples, the National Institute of Radiation Protection and Research (NIRPR), University of Ibadan, Nigeria, carried out the experiments to measure the soil samples’ radioactivity. The representative soil samples were dried at 105 °C in the oven for 6 h to eliminate any remaining moisture. A total of 500 g of homogenized soil was measured into a cylindrical-shaped plastic container measuring 7 cm in height and 6 cm in diameter.

Before gamma testing, the containers were hermetically sealed with adhesive tape for 30 days to allow for secular radioactive equilibrium between ^238^U and its short-lived progenies (AERB, 2003; Gbadamosi et al., 2018). An array of potential isotopes and gamma energies was matched by the computer application Maestro window, which was linked to the detector. On all the four sides and on top, a lead layer 10 cm thick and 15 cm thick protected the detector. The energy resolution of 2.0 keV and the relative efficiency of 33% at 1.33 MeV were achieved within the system utilizing a 18,000-s counting time. IAEA 315, the International Atomic Energy Agency, provided standard sources for calibration detector (IAEA, 2003). Measurements were conducted with reference and soil samples considering background radiation. The activity concentrations of ^40^K, ^232^Th, and ^238^U were discovered by ongoing spectrum research (Jibiri et al., 2014). The peak values for ^40^K, ^238^U, and ^232^Th are 1460 keV (^40^K), 1764.5 keV (^214^Bi), and 2614.5 keV (^208^Ti), respectively, in order to calculate the activity levels (Bq kg^−1^) (the peaks for ^238^U and ^232^Th are associated with their decay products (^214^Bi and ^208^Ti, respectively)). According to Jibiri et al. (2014) and Gbadamosi et al. (2018), Eq. 1 was used to calculate the activity concentration (Cs) of the radionuclide after deducting the decay adjustment factor.

1 $$C_s=\frac{R_{net}}{\varepsilon\gamma\times Y\gamma\times Ms\times Ct}$$

where Ms is the sample mass, Yγ is the yield of the gamma ray at a specific energy, Ct is the total counting time, Rnet is the net peak area of a peak at energy, and εγ is the detector efficiency for a γ-energy of interest. The minimum detectable activity (MDA) of every radionuclide was calculated for the same counting period as the soil samples. It was determined to be 1.8 Bqkg^−1^ for ^40^K, 0.7 Bq kg^−1^ for ^232^Th, and 0.37 Bq kg^−1^ for ^238^U (Eq. 2).

2 $$LLD=4.65\lfloor C_b/t_b\rfloor^2\times f$$

the background counting time (t_b_) is measured in seconds, the net background count in the subsequent peak is represented by C_b_, and the factor f is used to convert counts per second (cps) to activity concentration (Bq kg^−1^). Utilizing the known radionuclide activity ^40^K (578.4 keV), ^238^U (609 keV), and ^232^Th (911 keV) as a reference standard source allowed for additional calibration of the detector’s efficiency. A total of 17.3 Bq kg^−1^, 5.1 Bq kg^−1^, and 5.0 Bq kg^−1^ were the corresponding detection limits for ^40^K, ^238^U, and ^232^Th (Gbadamosi et al., 2018).

#### Radiological and health hazard parameters

The calculated total activity concentration of radionuclides does not accurately indicate health issues because to the unequal distribution of anthropogenic increased NORMs (^232^Th, ^40^K, and ^238^U) identified in quarry soil samples. Thus, it is necessary to estimate the radiological dangers brought on by the NORMs in the samples. To precisely quantify the risks faced by the population, radiation hazard indices were computed based on the activity concentrations of the discovered NORMs in the quarry soil at different regions. The absorbed dose rate (D_R_), annual gonadal dose equivalent (AGDE), radium equivalent (Ra_eq_), and excess lifetime cancer risk (ELCR). The activity utilization index (AUI), the H_int_ and H_ext_ external and internal hazard indices, the I_γr_ level index for γ-radioactivity, and the annual effective dose equivalent (AEDE) outdoor and (AEDE) indoor. All parameters related to health and radiological hazards were statistically analyzed using SPSS 21.0.

Absorbed dose rate

The absorbed dose rate (D_R_) from gamma radiation in open air at a height of 1 m above the ground surface, assuming a uniform distribution of naturally occurring radionuclides (^238^U, ^232^Th, and ^40^K), was calculated using the equation provided by UNSCEAR (2000). 

3 $$The\;absorbed\;dose\;rate\;(D_R)nGyh^{-1}=0.462C_U+0.604C_{Th}+0.0417C_K$$

C_U_, C_Th_, and C_K_ represent the mean activity concentrations of ^238^U, ^232^Th, and ^40^K in Bq kg^−1^, respectively.

Radium equivalent

4 $$Ra_{eq}(Bqkg^{-1})=(C_U+1.43C_{Th}+0.077C_K)$$

Raeq summarizes the weighted uniform activities of ^232^Th, ^238^U, and ^40^K in a sample, assuming that 370 Bq kg^−1^ of ^238^U, 259 Bq kg^−1^ of ^232^Th, and 4810 Bq kg^−1^ of ^40^K produce the same gamma radiation dosage (Eq. 4) (Qureshi et al., 2014).

Hazard indices

The outdoor and indoor radiation dangers are represented by the H_ext_ and H_int_ indices. The outdoor hazard index (H_ext_) is a crucial tool in determining if any material used in the construction of houses is safe from a radiological point of view, and it can be used to regulate indoor exposure to ^222^Rn and its radioactive offspring (Eqs. 5 and 6) (Isinkaye & Oyedele, 2014).

5 $$H_{ext}=C_U/370+C_{Th}/259+C_K/4810\leq<span class='crossLinkCiteEqu'>1</span>$$

6 $$H_{int}=C_U/185+C_{Th}/259+C_K/4810\leq<span class='crossLinkCiteEqu'>1</span>$$

The average activity concentrations of ^238^U, ^232^Th, and ^40^K in Bqkg^−1^ are represented by the symbols C_U_, C_Th_, and C_K_. For the purpose of building construction, the radiation risks associated with soils, or any other environmental matrix, must be deemed low if the index values are less than unity.

Annual effective dose equivalent

The annual effective dose equivalent consists of two types: the annual outdoor effective dose (AEDEoutdoor) and the annual indoor effective dose (AEDEindoor). To calculate the annual effective dose equivalent (AEDE), a conversion factor of 0.7 Sv Gy^−1^ was applied to convert the absorbed dose rate in air (nGy h^−1^) to the effective dose, using outdoor and indoor occupancy factors of 0.2 and 0.8, respectively. The radiological impact of the obtained results is ascertained using AEDE (Eqs. 7 and 8) (UNSCEAR, 2000). 

7 $$AEDE_{outdoor}=D_R\times8766h\times0.7Sv/Gy\times0.2\times10^{-3}$$

8 $$AEDE_{indoor}=D_R\times8766h\times0.7Sv/Gy\times0.8\times10^{-3}$$

Annual gonadal dose equivalent

The annual gonadal dose equivalent (AGDE) measures the genetic significance of the yearly γ-dose received by individuals through reproductive organs such as the gonads, bone marrow, and other reproductive cells. The AGDE due to the specific activity of 238U, 232 Th, and 40 K was calculated using the following equation (Gbadamosi et al., 2018).

The AGDE due to the specific activities of ^238^U, ^232^Th, and ^40^K was calculated using Eq. AGDE = + 9 (Caridi et al., 2021).

9 $$AGDE=3.09C_U+4.18C_{Th}+0.314C_K$$

Gamma level index

10 $$I_{\gamma r}=C_U/300+C_{Th}/200+C_K/3000\leq<span class='crossLinkCiteEqu'>1</span>$$

The absorbed gamma dose rate of 0.3 mSv year^−1^ is associated with Iγr ≤ 2, while the absorbed gamma dose of 1 mSv year^−1^ is associated with 2 < Iγr < 6 which suggests a lower radiological risk to soil and plants using Eq. 10 (Gbadamosi et al., 2018).

Activity utilization index

11 $$AUI=\lfloor C_U/50\rfloor f_U+\lfloor C_{Th}/50\rfloor f_{Th}+\lfloor C_K/500\rfloor f_K\leq<span class='crossLinkCiteEqu'>2</span>$$

AUI is used to estimate the potential radiation dose from a combination of naturally occurring radionuclides like thorium (^232^Th), radium (^238^U), and potassium (^40^K) in soil, representing the combined air dose rate from these elements. A value below 1 indicates a relatively low radiation exposure risk. The average activity concentration of ^238^U, ^232^Th, and ^40^K in Bq kg^−1^ in soil represents C_U_, C_Th_, and C_K_ respectively. The fractional contributions to the γ-radiation, which constitutes the treatment rate, of the actual radionuclides ^238^U, ^232^Th, and ^40^K are f_U_ (0.462), f_Th_ (0.604), and f_K_ (0.0417), respectively. This was calculated using Eq. AUI = + + 10 (Isinkaye & Emelue, 2015).

Exposure rate

The exposure rate (ER) is the amount of gamma radiation exposure that individuals might experience from a given source, typically measured in microsieverts per hour (µSv/h) or microrems per hour (µRh/h). Equation 12 was used to determine the quarry soil samples’ exposure rate to gamma radiation (UNSCEAR, 2000).

12 $$ER(\mu Rh^{-1})=1.90\;C_U+2.82\;C_{Th}+0.179\;C_K$$

Excess lifetime cancer risk

This provides an individual’s lifetime cancer risk based on anticipated radionuclide intakes and consequent exposures. The ELCRoutdoor is calculated using the following Eq. 13 (Ramasamy et al., 2014). 

13 $$ELCR=AEDE_{outdoor}\times DL\times RF$$

where AEDEoutdoor, DL, and RF are the annual outdoor effective dose equivalent, life expectancy (70 years), and the risk factor (Sv^−1^) for fatal cancer per Sievert. For stochastic effects, ICRP 60 uses a value of 0.05 for the public (ICRP, 2007).

## Results and discussion

### Activity concentration of radionuclides in the soil samples of different districts

Three naturally occurring radionuclides’ activity concentrations (^238^U, ^232^Th, and ^40^K) were determined in the different quarry soils of some populated district areas (Odeda, Ijebu Ode, and Ajebo) within Ogun state Southwest, Nigeria, and are tabulated (Table 1) and were compared with previous studies in Nigeria, India, and China (Table 2). The average activity concentration varies depending on the area, and this has been linked to the environment’s chemicals and mineralogical features displaying wide fluctuations (Issa et al., 2013).

**Table 1:** The activity concentration (*n* = 10) of the radionuclides in the quarry soil sample

Site	K- 40 (Bq/kg)	U- 238 (Bq/kg)	Th- 232 (Bq/kg)
SHG	2056.4 ± 359.4	55.4 ± 24.9	150.1 ± 73.4
SKY	967.0 ± 231.5	50.5 ± 13.7	124.1 ± 51.0
SAD	1046.3 ± 386.5	40.3 ± 11.4	179.7 ± 95.6
ALA	76.8 ± 44.8	3.2 ± 1.8	7.7 ± 5.5
24H	1262.5 ± 922.0	53.9 ± 12.6	244.4 ± 89.8
ABL	145.0 ± 66.8	5.1 ± 1.3	11.1 ± 7.4
HOY	94.7 ± 24.2	3.6 ± 1.9	5.2 ± 3.9
LAB	488.0 ± 110.7	17.5 ± 2.0	29.8 ± 3.0
SAF	2647.9 ± 179.4	48.2 ± 18.9	18.6 ± 8.0
RAT	2552.9 ± 137.2	45.5 ± 10.0	7.4 ± 2.6
ODE control	97.8 ± 1.7	0.7 ± 0.0	0.9 ± 0.1
IJB control	102.3 ± 1.8	4.8 ± 0.1	4.3 ± 0.2
AJB control	29.5 ± 0.9	1.9 ± 0.1	3.9 ± 0.2
Mean	1133.7 ± 498.8	32.3 ± 19.6	77.8 ± 37.2
Kurtosis	− 1.2	− 1.9	− 0.7
Skewness	0.5	− 0.5	0.9
Minimum	76.8 ± 44.8	3.2 ± 1.8	5.2 ± 3.9
Maximum	2647.9 ± 179.4	55.4 ± 24.9	244.4 ± 89.8
World average	420	33	45

**Table 2:** Comparison of the radioactive activity levels in the soil with earlier research on bitumen, quarry soils, granite, and sediments worldwide

Location	Type of sample	K- 40 (Bq/kg)	U- 238 (Bq/kg)	Th- 232 (Bq/kg)	Reference
Bengal, India	Sediment	382.00	11.40	41.20	Karuppasamy et al., 2024
Asa, Nigeria	Granite soil	441.06	11.51	15.42	Orosun et al., 2020
Ishiagu, Nigeria	Quarry soil	141.30	22.50	13.70	Nduka et al., 2022
Moba, Nigeria	Soil and rock	222.20	30.40	3.31	Isinkaye et al., 2023
Gulmit, Northern Pakistan	Sediment	173.96	11.65	21.37	Qureshi et al., 2014
Edo, Nigeria	Surface soil	1.41	4.85	30.19	Popoola et al., 2019
Ondo, Nigeria	Tar sand	46.48	24.13	20.11	Isinkaye et al., 2018
IleIfe, Nigeria	Mining soil	270.14	12.14	23.23	Oluyide et al., 2019
Bayanwula, China	Surface soil	923	26	29	Bai et al., 2017
Odeda, Nigeria	Quarry soil	2056.44	55.42	150.10	Present study
Ajebo, Nigeria	Quarry soil	1262.40	53.97	244.36	Present study
Ijebu Ode, Nigeria	Quarry soil	2647.88	48.21	29.83	Present study
Global average	Soil and rock	420	33	45	UNSCEAR, 2000

In Odeda area, the activity concentration of ^238^U, ^232^Th, and ^40^K ranged between 3.2 and 55.4, 7.7 and 179.7, and 76.8 and 2056 Bq/kg respectively. The level of potassium activity was higher in the soil from the quarry due to the presence of micronutrients such as iron (Fe), copper (Cu), and manganese (Mn) (Karuppasamy et al., 2024). It was evident from the analysis that SHG site samples have the highest concentration of ^238^U and ^40^K while SAD site has the highest level of ^232^Th. ALA site have the lowest level of analyzed activity concentration of radionuclides in all the sites in Odeda area. All quarry sites have concentrations of ^40^K that are two to four times higher than the global average of 420 Bq/kg, when compared to the recommended limit (UNSCEAR, 2000), except for the ALA site. The results of Adel Gawad et al. (2024), where ^40^K is 992.3 Bq/kg, are comparable to this. Furthermore, the concentration of ^238^U is 1.5 times greater than the global average of 33 Bq/kg (UNSCEAR, 2000) in every site but the ALA site, where the average is 3.2 Bq/kg. The world average and recommended limit of ^232^Th (45 Bq/kg) (UNSCEAR, 2000) is 3–4 times lower than the average concentrations in all sites except in ALA site. The control site in Odeda area has values lower than the world average (Table 1). All the quarry sites in this region are contaminated because they have activity concentrations values of NORMs to be above the world recommended limit except in ALA site. Activity concentration levels are increasing at all sampling locations, with values falling between ^238^U, ^232^Th, and ^40^K (Table 1). Significant differences in the standard deviation values, which indicate an estimate of the geographical distribution of radionuclides inside the different quarry sites, point to a major variance in the activity concentrations. Also, it illustrates that the impact of radioactive buildup in the soil at the sites is caused by physical and geochemical processes (Jegede et al., 2019). According to Table 1, the activity concentration of NORMs in the Ajebo area varies from 3.6 to 53.9 Bq/kg, 5.2 to 244.4 Bq/kg, and 94.7 to 1262.5 Bq/kg for ^238^U, ^232^Th, and ^40^K respectively.

It was evident from the results that 24H site has the highest average concentration for all the radionuclides while HOY site has the lowest concentration. The average activity concentrations in all quarry sites in this area have values that is 2–4 times more than the world average (420 Bq/kg) for ^40^K except in ABL and HOY site. It has been determined that quarry soil are basically a mixture of granitic igneous and sedimentary rock, and they serve as a host for NORMs, hence the reason for the high activity concentration in some soils (Ofomola et al., 2023).

All of the area sites, apart from the 24H site, exhibited average levels of concentrations of ^238^U and ^232^Th that were 1.5 times and 5 times higher, respectively, than the global averages of 33 Bq/kg and 45 Bq/kg. In all sites, the increasing level of activity concentration in this area is in the range ^238^U < ^232^Th < ^40^K (Table 1). The recommended limit for activity concentration worldwide is exceeded by the control site located in the Ajebo area. In Ijebu Ode area quarry sites, ^238^U, ^232^Th, and ^40^K activity concentrations ranged from 48.2 to 45.5 Bq/kg, 7.4 to 18.6 Bq/kg, and 2552.9 to 2647.8 Bq/kg, respectively. The sites with the highest and lowest concentrations, respectively, among all examined NORMs are SAF and RAT. The global average value of 45 Bq/kg is 3–6 times greater than the concentration of ^232^Th activity, and the levels of ^40^K and ^238^U activity are 1.5 and 6 times higher, respectively, than the global average values of 420 Bq/kg and 33 Bq/kg (UNSCEAR, 2000). In every site, the radionuclide activity concentration rises in the range ^232^Th < ^238^U < ^40^K. The activity concentration at the control site for the quarry sites under evaluation in the Ijebu Ode area is below the global average limit. The main cause of the significant variations in the NORM activity concentrations in each sample from the quarry site sample is the degree of activity in the quarry, which is dependent on the chemical, physical, and geochemical properties of the accumulated wastes at the locations. Previous studies have linked the mobility and solubility of U(VI)O^2+^_2_ to the high level of activity concentration of ^238^U found in 70% of the total samples in this investigation (Skoko et al., 2017). The high concentration of ^232^Th and ^40^K can also be associated with the loamy soil that has mixed with the quarry soil, as well as the aggregation of various mineral components in that soil and the increased rate of application of fertilizers rich in phosphorus, potassium, and nitrogen (NPK) for various agricultural uses (Gbadamosi et al., 2018). The high amount of potassium is also linked to the findings of Runsheng et al. (2023), who examined the geochemistry of Zn-Pb deposits in the Sichuan-Yunnan-Guizhou Triangle area, and provided insight into how mining and geological processes influence the concentration of elements like potassium in environments subject to human activities. The activity concentrations of naturally occurring radioactive materials (NORMs) vary across quarry sites within the same geographic region due to differences in geological composition, mineralogy, and soil formation processes (Gitonga et al., 2024). Factors such as rock type, weathering, groundwater movement, and leaching affect the mobility and distribution of radionuclides like uranium (^238^U), thorium (^232^Th), and potassium-40 (^4^⁰K). Additionally, quarrying methods, depth of excavation, and human activities can expose or concentrate NORMs differently, leading to significant site-specific variations (Asere & Sedara, 2020).

### Radiological hazard parameters for the radionuclides in different district areas

#### Absorbed dose rate

Tables 3–5 display the radioactive risk characteristics for the quarry sites in the three district areas (Ijebu, Odeda, and Ajebo) under examination in Ogun State. The radiation absorbed dose rate generated by terrestrial gamma ray activities at a level 1 m above the ground is calculated using the activity concentrations of ^238^U, ^232^Th, and ^40^K. In the quarries located in the Odeda area, the absorbed dose rate has a mean value of 130.20 nGy/h and a range of 3.03–282.35 nGy/h (Table 3). This zone’s mean dose rate is more than twice as high as the global average, which is at 57 nGy/h (UNSCEAR, 2000). In these zones, the absorbed dose rate is higher than the acceptable limit in 75% of samples collected from twenty (20) different locations (Table 3). The mean average in the Ijebu Ode zone was found to be 137.93 nGy/h, with a minimum of 118.06 and a high of 157.29 nGy/h (Table 4). Every sample in this region had a value that was higher—nearly three times higher—than the global average, and the absorbed dose rate for the Ajebo zone ranged from 3.64 to 339.92 nGy/h, with a mean value of 73.88 (Table 5). A higher percentage (65%) of the samples taken in various locations in these areas have a value lower than the world average (57 nGy/h). Owing to the NORMs in the quarry rock and soils, the environment is ideal for low level gamma radiation exposure. The quarry workers and other local farmers’ prolonged exposure to the low-level radiation over time may have long-term adverse health impacts. People exposed to radiation may experience effects on their tissues and organs (Ofoma et al., 2023).

**Table 3:** The mean values of the activity concentration and radiological parameters in samples of quarry soil (*n* = 40) and control (*n* = 2) in all the sites around Odeda area

	Mean	Control	Minimum	Maximum
^40^K (Bq/kg)	1036.6 ± 269.8	97.8 ± 1.7	76.8 ± 44.7	2056.4 ± 359.4
^238^U (Bq/kg)	37.4 ± 5.8	0.7 ± 0.0	3.2 ± 1.8	55.4 ± 24.9
^232^Th (Bq/kg)	115.4 ± 45.8	0.9 ± 0.1	7.7 ± 5.5	179.7 ± 95.6
D_R_ (nGy/h)	130.20	4.98	3.03	282.35
R_aeq_ (Bq/kg)	282.22	9.60	5.88	614.92
H_ext_	0.76	0.03	0.02	1.66
H_int_	0.86	0.03	0.02	1.85
AEDE out (µSvy^−1^)	159.78	6.11	3.72	346.52
AEDEin (µSvy^−1^)	639.13	24.46	14.89	1386.06
ELCR × 10^−3^	0.56	0.02	0.01	1.21
AGDE (µSvy − ^1^)	923.36	36.89	22.37	2003.03
Iγr	1.05	0.04	0.02	2.28
AUI	1.83	0.03	0.02	4.61
ER(µRh^−1^)	581.00	21.54	13.10	1267.67

**Table 4:** The mean values of the activity concentration and radiological parameters in samples of quarry soil (*n* = 20) and control (*n* = 2) in the sites around Ijebu Ode area

	Mean	Control	Minimum	Maximum
^40^K (Bq/kg)	2600.42 ± 587.74	102.27 ± 1.76	2354.12 ± 132.41	2957.66 ± 163.51
^238^U (Bq/kg)	46.85 ± 12.53	4.82 ± 0.10	35.25 ± 6.80	80.32 ± 10.8
^232^Th (Bq/kg)	12.99 ± 4.79	4.27 ± 0.20	2.99 ± 0.28	29.25 ± 5.30
D_R_ (nGy/h)	137.93	8.91	118.06	157.29
R_aeq_	265.66	18.57	224.68	304.82
H_ext_	0.73	0.05	0.61	0.82
H_int_	0.84	0.06	0.71	1.01
AEDE_out_ (µSvy^−1^)		10.94	144.88	193.03
AEDE_in_ (µSvy^−1^)	677.10	43.74	579.53	772.12
ELCR × 10^−3^	0.59	0.04	0.51	0.68
AGDE (µSvy^−1^)	1015.61	64.12	872.63	1159.89
Iγr	1.09	0.07	0.93	1.25
AUI	0.81	0.10	0.59	1.08
ER(µRh^−1^)	591.13	39.33	504.19	678.88

**Table 5:** The mean values of the activity concentration and radiological parameters in samples of quarry soil (*n* = 40) and control (*n* = 2) around Ajebo area

	**Mean**	**Control**	**Minimum**	**Maximum**
^40^K (Bq/kg)	497.55 ± 37.78	29.54 ± 0.95	41.54 ± 1.12	2814.34 ± 3.88
^238^U (Bq/kg)	20.05 ± 12.79	1.95 ± 0.06	0.47 ± 0.03	75.80 ± 14.52
^232^Th (Bq/kg)	72.63 ± 26.31	3.9 ± 0.19	0.34 ± 0.19	430.63 ± 48.47
D_R_ (nGy/h)	73.88	4.49	3.64	339.92
R_aeq_	162.21	9.80	6.90	739.46
H_ext_	0.44	0.03	0.02	2.00
H_int_	0.49	0.03	0.02	2.14
AEDEout (µSvy^−1^)	90.66	5.51	4.47	417.16
AEDEin (µSvy^−1^)	362.66	22.03	17.86	1668.64
ELCR × 10^−3^	0.32	0.02	0.02	1.46
AGDE (µSvy^−1^)	521.76	31.60	27.10	2419.17
Iγr	0.60	0.04	0.03	2.76
AUI	1.10	0.07	0.01	5.76
ER (µRh^−1^)	331.96	19.99	15.66	1531.47

#### Radium equivalent

**Fig. 2:**
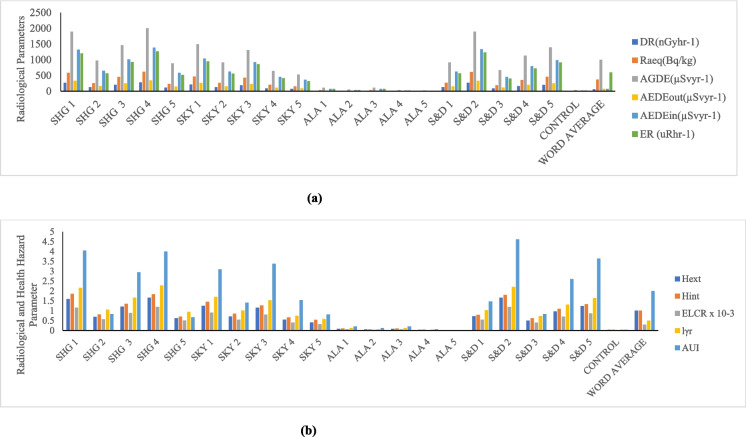
** a** Radiological and health hazard parameters of Odeda District for D_R_, Ra_eq_, AGDE, AEDE, and ER. **b** Radiological and health hazard parameters of Odeda District H_ext_, H_int_, ELCR, I yr, and AUI.

**Fig. 3:**
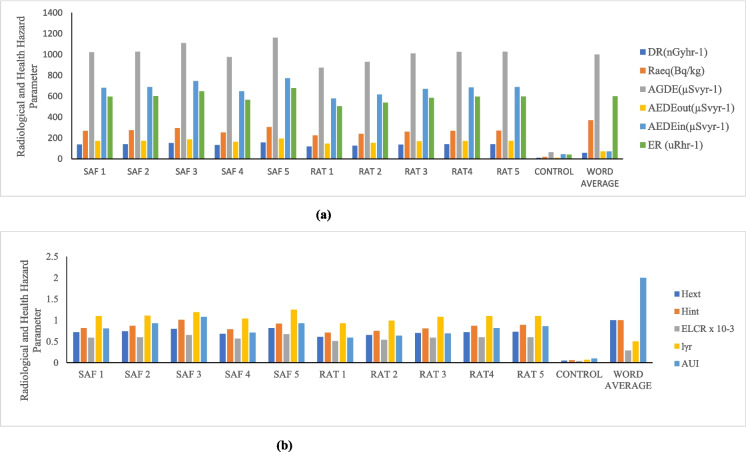
** a** Radiological and health hazard parameters of Ijebu District D_R_, Raeq, AGDE, AEDE, and ER. **b** Radiological and health hazard parameters of Odeda District Hext, Hint, ELCR, Iyr, and AUI.

According to Table 3, the computed value for Odeda ranged from 5.88 to 614.92 Bq/kg, with an average value of 282.22 Bq/kg. The average value obtained is lower than the world average limit of 370 Bq/kg (Fig. 2). This was in tandem with the findings of Azeem et al. (2024). Less than 35% of all the samples taken from this location are higher than the recommended limit. The radium equivalent of sample taken from Ijebu Ode area gave an average value of 265.68 with a minimum value of 224.68 and maximum value of 304.82 (Fig. 3). This was close to the findings of Zakaly et al. (2024) (93.6–209.9 Bq/kg). Table 5 displays the mean value and range for the Ajebo area, which were 162.2 Bq/kg and 6.90–739.46 Bq/kg, respectively. While the global suggested limit for radium equivalent is exceeded by 15% of the samples collected from those locations, the average value of radium equivalent in this area is still lower than that limit. This is demonstrated by the table data. Based on these results from the sampling points, almost all the soil samples in the different district areas showed a low radium equivalent value and therefore implies that the soil of the quarries could be made available and used without any hindrance for building and construction purposes in the environment.

#### Internal radiation hazard index and external hazard index

Table 4 displays the estimated internal hazard index values for the Odeda area, which range from 0.02 to 1.85, with an average value of 0.86. Although 40% of the samples taken from this area have values higher than the global average of unity (1), the mean value (as shown in Fig. 2) is less than unity (1), the world average recommended unit. This is in tandem with the findings of Ofomola et al. (2023) regarding their value of 0.51.

The control sample for this area showed a value of 0.03. In Ijebu Ode area, the internal radiation hazard ranged from 0.71 to 1.0.2 with an average value of 0.84 (Table 4). This is below the recommended unit. Except one sample in SAF site, all others from this location are below the recommended world limit. The control sample for the Ijebu Ode region also gave a calculated value (0.06) lower than the world limit (Fig. 3). The average internal hazard index value of soil sample taken from Ajebo area is 0.49 with the minimum value of 0.02 and maximum value 2.14 (Table 5). The mean value is lower than the world recommended limit of 1, and also that of Azeem et al. (2024) with a value of 0.46. Fifteen percent of the samples taken from this area have a value higher than the world limit while the control sample has a value (0.03) which is lower than the world limit. The radionuclide radon- 222, which is known to be a progeny of ^226^Ra and poses threats to the respiratory organs when accumulated in higher amounts in the indoor air, will no longer be a threat to the environment because the soil samples taken from Ijebu Ode and Ajebo are more pristine and can be recommended for use because the internal hazard index is less than one.

According to Jegede et al. (2019), the external hazard is a dose anterior that is used to determine the external dosage to humans in a house created with such materials as well as to evaluate the suitability of any material intended for use in constriction. According to Table 3, the Odeda area’s external hazard indices had a mean value of 0.76 and a range of 0.02–1.66. While 35% of the sample obtained in these locations has a value higher than the world limit, and the control sample has a value (0.03) lower than the world average limit, the average value is below the UNSCEAR (2000) recommended limit of 1. Table 4 displays the mean value of Ijebu ode, which is 0.73 on average, with a maximum value of 0.82 and a minimum value of 0.61. The global average limit of one is exceeded by this. The control sample, which has a value of 0.03, is among the samples from these regions that are all below the suggested limit. As shown in Table 3, the average value of the Ajebo area external danger index is 0.49, which is twice below the word average limit (UNSCEAR, 2000). The index ranges from 0.02 to 2.00. Fifteen percent more samples above the acceptable threshold were collected overall from the Ajebo area. Some soil samples in Odeda and Ajebo exceed the globally recommended limit; as a result, the soil in these areas poses an internal radiological risk to workers and residents in the vicinity of the study site. This is because the radionuclides present in the soil sample have harmful effects on ionizing radiation.

**Fig. 4:**
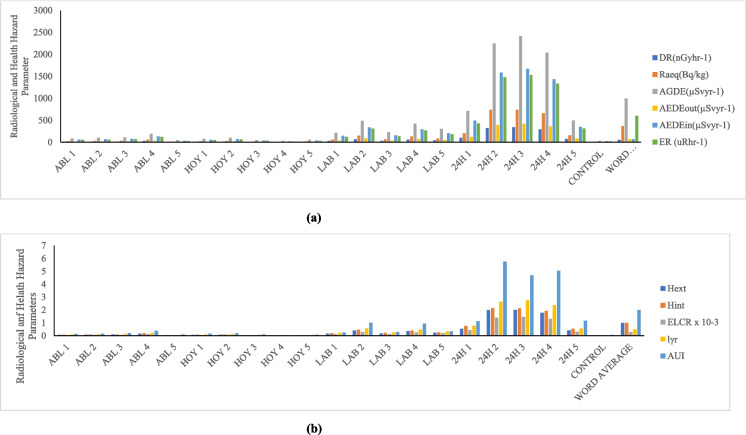
** a** Radiological and health hazard parameters of Ajebo District for D_R_, Ra_eq_, AGDE, AEDE, and ER. **b** Radiological and health hazard parameters of Odeda District for H_ext_, H_int_, ELCR, I_yr_, and AUI.

#### The annual effective dose equivalent (indoor and outdoor)

For the indoor value, it was shown (Table 3) that the mean value is 639.13 while the minimum is 14.89 with a maximum of 1386.06 for Odeda area. The Ijebu Ode area also showed a range value of 579.53–772.12 with an average value of 677.10 (Table 4) while the samples from Ajebo area have a mean value of 362.66 and a range 17.86–1668.64 (Table 5). The annual effective dose equivalent values (AEDEout) for Odeda area ranged from 3.72 to 346.52 with a mean value of 159.78 (Table 5). The average value is more than twice higher than the global average value of 70 (UNSCEAR, 2000) (Fig. 4). This is an indicator that a long-term exposure to the quarry soil has an adverse effect on the health such as cardiovascular, tissue degeneration, and cancer (Abdel-Gawad et al., 2024). The ALA site in that district area represents 25% of the samples taken in Odeda, and the values of the soil in that zone are less than the global average of 70. Also, the Ijebu Ode area has an average of 169.28 with a range value of 144.88–193.03 (Table 4). None of the samples collected in this location has a value below the globally advised control limit, and the average value is two times higher than the average value worldwide. Table 5 shows that the samples collected at Ajebo range in value from 4.47 to 417.16, with a mean of 90.66. This mean value exceeds the globally advised threshold. The values of 65% of the collected soil sample from the Ajebo region are below global thresholds.

#### Annual gonadal dose equivalent

The AGDE of each location was computed using the activity concentrations of ^238^U, ^232^Th, and ^40^K. In the Odeda region, a mean average of 923.36 was determined, with a range value of 22.37–2003.03 (Table 3). Of the samples taken from this region, the mean average was lower than the global average of 1000 µSvy^−1^ in 60% of the samples. With a value of 36.89, the control samples are much below the global limit. Samples in Ijebu Ode vary from 872.63 to 1159.89, with a mean value of 1015.61 (Table 4). This mean value is above the world recommended limit. Seventy percent of the sample are higher than the global limit. The control site value 64.12 is lesser than the world limit. The Ajebo area also has an average value of 521.76 with a minimum of 27.10 to maximum 2419.17 (Table 5). This average value for Ajebo is less than the world average value of 1000 (UNSCEAR, 2000) (Fig. 4). Seventy-five percent of the samples in these areas have values less than the world average limit. The site used for control gives a value of 31.60 which is also less than the world average.

#### Excessive lifetime cancer risk

The potential for an individual exposed to soil samples to develop malignant cells is known as the excessive lifetime cancer risk, and it is used to evaluate the carcinogenicity and mutagenicity effects of the soil samples. Radionuclides present in building and construction materials have been discovered to produce carcinogenic effects (Pradhoshini et al., 2025). This is due to the exposure to gaseous radionuclides, e.g., radon indoors, and an accumulation of these gaseous radionuclides over life time of an individual. The possibility of an individual working or living around a quarry site to accumulate this radionuclide over time thereby leading to cancer can be calculated using ELCR. The ELCR value of Odeda area was calculated as shown (Table 3). The average value is 0.56 × 10^−3^ with a range 0.01 × 10^−3^–1.20 × 10^−3^. The world average value of 2.9 × 10^−3^ is twice as high as the mean value. Azeem et al. (2024) (0.349 × 10^−3^), Shrestha et al. (2024) (1.2 × 10^−3^), and Abdel-Gawad et al. (2024) (1.86 × 10^−3^) have also found lower values than the global average. The world average is exceeded in 75% of the samples collected from this region. The value (0.02 × 10^−3^) obtained from this area’s control site is 13 times lower than the global average. Moreover, the samples evaluated in the Ijebu Ode region show a mean value of 0.59 × 10^−3^, a minimum of 0.51 × 10^−3^, and a high of 0.68 × 10^−3^ (Table 4). In this region, the ELCR value of every sample (100%) is 2–3 times lower than the global average (Fig. 3). The control value in this location is less than the global average, at 0.04. Table 5 displays the mean value of 0.32 × 10^−3^ for the Ajebo samples, ranging from 0.02 × 10^−3^ to 1.46 × 10^−3^. All of the samples from this location have mean values that are lower than the global average. The 24H samples are all five times lower than the global average. The control sample (0.02 × 10^−3^) is 15 times lower than the global average. The results clearly show that the Ijebu Ode samples are below the global limit (Fig. 3), which means that there is reduced chance of cancer for those who live or work near the quarry sites because they will not be exposed to the NORMs present. Also, the soil of these sites is good for construction, building, or agricultural purpose/remediation of the soil; therefore, people should be encouraged to use the soil in quarry sites found in this area, for the purposes listed.

#### Gamma level index

According to Table 3, the radionuclide gamma index level in Odeda soil ranges from 0.02 to 2.28, with an average value of 1.05. At this location, 55% of the sample is higher than the global average, indicating that the value is greater than the 0.5 world average limits. Also, the Ijebu Ode area has an average of 1.09 with a range of 0.93–1.25 (Table 4). These results showed 70% of the samples to be higher than the global limit recommendation of 0.5, but the control sample is lower than the recommended limit (0.07). A higher percentage (80%) of the sample gave a calculated value lower than the world limit. In Ajebo district area, the mean of the sample taken from this region is 0.60 which is lower than the global recommended limit. The findings of Shrestha et al. (2024) are like the world average value (0.5) as compared to all the district areas.

### Multivariate statistical analysis

**Fig. 5:**
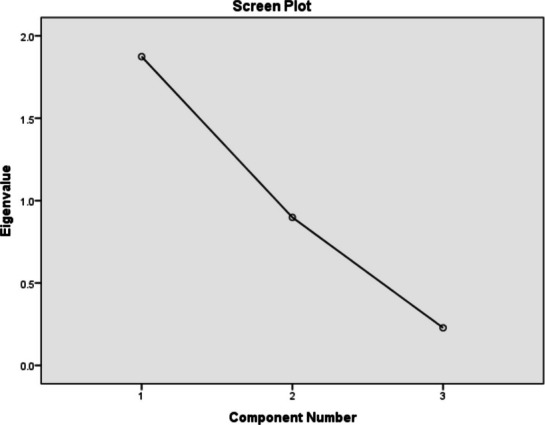
Screen plot showing the initial Eigen values of each component of the radionuclides in the study

Applying principal component analysis (PCA) and Pearson’s correlation to the activity concentration of naturally occurring radionuclide materials (NORMs) in quarry soils using the commercial statistical software package SPSS (version 21.0, Inc., Chicago IL) allowed for the valid conclusion to be made regarding the interrelation among the variables. The correlation coefficient matrix for radionuclides, Pearson product moment, is shown in Table 6. ^40^K and ^238^U (*r* = 0.6664) and ^232^Th and ^238^U (*r* = 0.482) showed significant associations at the *P* < 0.01 level. There were rather significant positive correlations discovered between ^40^K and ^238^U as well as between ^232^Th and ^238^U. There is little association between ^40^K and ^232^Th (*r* = 0.107). They come from the same sources, as evidenced by the tight link between ^238^U and ^40^K and ^232^Th and ^238^U. According to Zhuo et al. (2019), the first principal component (PCI) usually gives a better explanation about the largest part of the variation in the data set which is usually around 40%, which is placed along the direction of maximum variance, while the second component (PC2) is positioned orthogonally to the first PC and in the direction of the second largest variation, and that continues for the PC3, PC4, etc. With Kaiser-Meyerolkin (KMO) sample adequacy measurement and Bart-lett’s test of sphericity being appropriate and significant for the variables, principal component analysis was used to analyze the variables based on varimax orthogonal rotation. The screen plot displaying the initial Eigen values of NORMs (Fig. 5 and Table 7) is displayed, along with the rotated component matrix for NORMs (Table 8). In the quarry sites, only one component was extracted for the data for NORMs, and thus, component plots cannot be produced for the PCA analysis. The single component of ^238^U, ^40^K, and ^232^Th are 0.937, 0.777, and 0.613 respectively. Based on this, it is evident that all the radionuclides are from a single anthropogenic or lithogenic source.

**Table 6:** The correlation matrix of the naturally occurring radionuclides (NORMs) in this study

	K- 40	U- 238	Th- 232
K- 40	1.000		
U- 238	0.664	1.000	
Th- 232	0.107	0.0482	1.000

**Table 7:** The total variance of naturally occurring radionuclides (NORMs) in the study

	Initial Eigen value			Extraction sums of squared loadings		
	Total	% of variance	Cumulative %	Total	% of variance	Cumulative %
1	1.874	62.460	62.460	1.874	62.460	62.460
2	0.898	29.939	92.399			
3	0.228	7.601	100.000

**Table 8:** The contribution of statistically naturally occurring radionuclides (NORMs) variables examined in this study is shown using factor analysis (after varimax rotation)

Variables	Component 1
U- 238	0.937
K- 40	0.787
Th- 232	0.613

## Conclusion

The activity concentrations of naturally occurring radioactive materials (NORMs) in the quarry sites exhibit significant variation, which can be attributed to the distinct geological and geochemical properties of the rocks in each site. This variation is consistent with findings in Zhang et al. (2024), which demonstrate how human activities can influence the transport and distribution of solutes in river basins, showing that localized geological conditions play a critical role in determining the extent of contamination. Similarly, the geological composition of the quarry sites, including loamy soil interaction and mineral aggregation, is a significant factor contributing to the observed elevated levels of potassium –40 (^40^K) and thorium- 232 (^232^Th). The uranium –238 (^238^U) activity levels are notably high in 75% of the samples, a phenomenon attributed to the mobility and solubility of U(VI)O^2+^_2_, which is in line with the findings of Zhang et al. (2024), where human influence altered the natural transport behaviors of solutes. In this case, the high mobility of ^238^U points to the potential for increased radiological hazards in areas with similar geological conditions.

While some radiological parameters, such as absorbed dose rate (DR), Ir, Hint, AEDEoutdoor, and ELCRoutdoor, are below the global average, others exceed the recommended safety levels, indicating that residents and workers near the quarry sites may face significant radiation exposure risks. These exposures are associated with potential health concerns, including acute leucopenia, anemia, chronic lung diseases, and oral necrosis. In particular, the higher levels of radiation in the Odeda quarry samples emphasize the urgent need for intervention.

The principal component analysis (PCA) and the correlation coefficient matrix reveal a strong correlation between NORMs, suggesting that they originate from the same source and are influenced by both anthropogenic and lithogenic activities. The results reinforce the conclusion that these pollutants are interconnected, with human activity likely exacerbating their distribution.

The levels of NORMs detected in the quarry soils render them unsuitable for use in industrial and ecological applications. For instance, industries such as agriculture, where soil quality is paramount for crop production, could suffer from reduced yields and contamination of food products. Additionally, the construction industry could be at risk, as the contaminated soil could lead to radiological exposure during material processing or transport. The mining and environmental restoration industries could also face significant challenges, as the widespread presence of NORMs could lead to soil remediation complications and higher environmental cleanup costs.

Therefore, it is recommended that the use of soil from these quarry sites be discouraged for industrial and ecological purposes. Industries that rely heavily on soil quality, such as agriculture and construction, should avoid utilizing quarry-derived soil. Public awareness campaigns must be launched to educate residents and workers on the health risks associated with NORM exposure, urging them to take protective measures. Governments should implement strict monitoring protocols and enforce regulations to prevent further environmental contamination from NORMs and protect public health. Regular scientific evaluations and assessments of the impact of NORMs on the surrounding communities are essential for mitigating the long-term risks associated with these materials.

## Data Availability

No datasets were generated or analysed during the current study.

## Declarations

All authors have read, understood, and have complied as applicable with the statement on “Ethical responsibilities of Authors” as found in the Instructions for Authors.
